# Draft Genome Sequence of Sphingomonas sp. Strain Ant20, Isolated from Oil-Contaminated Soil on Ross Island, Antarctica

**DOI:** 10.1128/genomeA.01309-14

**Published:** 2015-01-08

**Authors:** Sandra Ronca, Aline Frossard, Leandro D. Guerrero, Thulani P. Makhalanyane, Jackie M. Aislabie, Don A. Cowan

**Affiliations:** a Centre for Microbial Ecology and Genomics, University of Pretoria, Pretoria, South Africa; b Landcare Research, Hamilton, New Zealand

## Abstract

Here, we present the draft genome of Sphingomonas sp. strain Ant20, isolated from oil-polluted soil near Scott Base, Ross Island, Antarctica. The genome of this aromatic hydrocarbon-degrading bacterium provides valuable information on the microbially mediated biodegradation of aromatic compounds in cold-climate systems.

## GENOME ANNOUNCEMENT

Antarctic exploration has led to localized, although significant, impacts on the environment. For example, fuel spills can have substantial environmental impacts on Antarctic ice-free soils through the accumulation of aliphatic and aromatic compounds (1, 2). The protocol for contaminated-soil recovery requires that soil remediation not result in greater environmental impact than that with the “do-nothing” approach (3). To achieve this goal, bioremediation is viewed as an alternative technology for cold-climate soils (4, 5). As the Antarctic Treaty prevents the introduction of foreign organisms into the continent, the isolation of indigenous bacterial strains with bioremediation capabilities is the preferred approach. The Gram-negative Sphingomonas sp. strain Ant20, isolated from oil-contaminated soil near Scott Base, Antarctica, was observed to utilize aromatic hydrocarbons, such as phenanthrene and 1-methylnaphthalene, as growth substrates (6). We sequenced the genome of this bacterial strain to unravel its genetic potential as a biodegrader.

Sphingomonas sp. Ant20 was grown on Reasoner’s 2A (R2A) medium at 15°C. DNA was extracted according to a modified protocol from Miller and colleagues (7), and the genome was sequenced on an Ion Torrent PGM sequencer (318 Chip) with 400-bp chemistry (Life Technologies) at the DNA Sequencing Facility of the University of Pretoria, Pretoria, South Africa. After quality filtering using in-house scripts, 2,403,622 reads with an average size of 241 bp were assembled using MIRA 4.Orc4 (8). The connections between the resulting contigs with a minimum length of 1,000 bp were checked on cytoscape 3.x (9) and assembled using GAP5 (10). The final draft genome contained 299 contigs, with a mean size of 15,009 bp and a maximum length of 191,481 bp. The total genome size was 4,487,680 bp, with a mean G+C content of 65.6% and an average coverage of 132×. The genome was annotated using the NCBI Prokaryotic Genome Annotation Pipeline (http://www.ncbi.nlm.nih.gov/genome/annotation_prok) and Rapid Annotations using Subsystems Technology (RAST) (11), which identified 4,580 protein-coding sequences (1,374 of annotated function and 3,206 hypothetical proteins). A comparison of the 16S rRNA gene sequences on NCBI indicated 96% identity with *Sphingomonas taxi* strain ATCC 5566 (GenBank accession no. CP009571.1). Annotation in RAST showed that interesting genes/operons were present in the Sphingomonas sp. Ant20 genome, including genes related to resistance to antibiotics and toxic compounds, such as genes encoding metal efflux pump proteins (e.g., for arsenic, cobalt-zinc-cadmium, and copper). Several stress-related proteins were identified, including cold and heat shock, oxidative, and carbon starvation proteins. Moreover, a number of proteins involved in various aromatic compound degradation pathways were found, including pathways for quinate, benzoate, beta-ketoadipate, *N*-heterocyclic aromatic, and aromatic amine compounds. However, no protein was predicted for phenanthrene and naphthalene degradation, despite the fact that Ant20 strain has been shown experimentally to degrade these compounds (6).

The analysis of the draft genome of Sphingomonas sp. Ant20 thus indicates that this organism may be considered a candidate for bioremediation in cold soils. In addition, the availability of previously annotated genomes of bacterial strains also isolated from oil-contaminated soils in Antarctica will allow comparative genomic studies and the comprehension of factors underlying the ability to degrade aromatic compounds.

### Nucleotide sequence accession numbers.

This whole-genome shotgun project has been deposited in DDBJ/ENA/GenBank under the accession no. JRVI00000000. The version described in this paper is version JRVI01000000.
